# The Evolving Roles of Nurses Providing Care at Home: A Qualitative Case Study Research of a Transitional Care Team

**DOI:** 10.5334/ijic.5838

**Published:** 2022-01-20

**Authors:** Wei Ting Chen, Hong-Gu He, Yeow Leng Chow

**Affiliations:** 1 Advanced Practice Nurse, Division for Central Health, Tan Tock Seng Hospital, SG; 2 Alice Lee Centre for Nursing Studies, Yong Loo Lin School of Medicine, National University of Singapore, Singapore; 3 National University Health System, SG

**Keywords:** delivery of health care, integrated, home nursing, community health nursing, role

## Abstract

**Purpose:**

To examine the roles of transitional care nurses in an integrated healthcare system and how the integrated healthcare system influences their evolving roles.

**Background:**

Transitional care teams have been introduced to enable the seamless transfer of patients from acute-care to the home settings. A qualitative case study of the transitional care team was conducted to understand the changing roles of these nurses in an integrated Regional Health System (RHS) in Singapore.

**Methods:**

A hospital transitional team of an integrated RHS was studied. Purposive sampling was used. Non-participant observations and follow-up interviews were conducted with four nurses. Data were triangulated with the interviews of two managers and three healthcare professionals, and the analysis of documents. Within-case thematic analysis was carried out.

**Results:**

Three themes were identified: ‘Coming together to meet the needs of all’; ‘Standing strong amidst the stormy waves’; and ‘Searching for the right formula in handling complexity’. These themes have explained on the atypical roles taken on by nurses in their attempts to close the gaps and meet the patients’ needs. Various factors influencing the evolving roles were revealed.

**Conclusion:**

The roles of nurses have ‘emerged differently’ from their traditional counterparts. Various nursing roles have been undertaken to facilitate care integration. The findings emphasised the important balance between formal structural practices and informal processes in facilitating and supporting the nurses in their role development.

## Introduction

**Figure 1:**
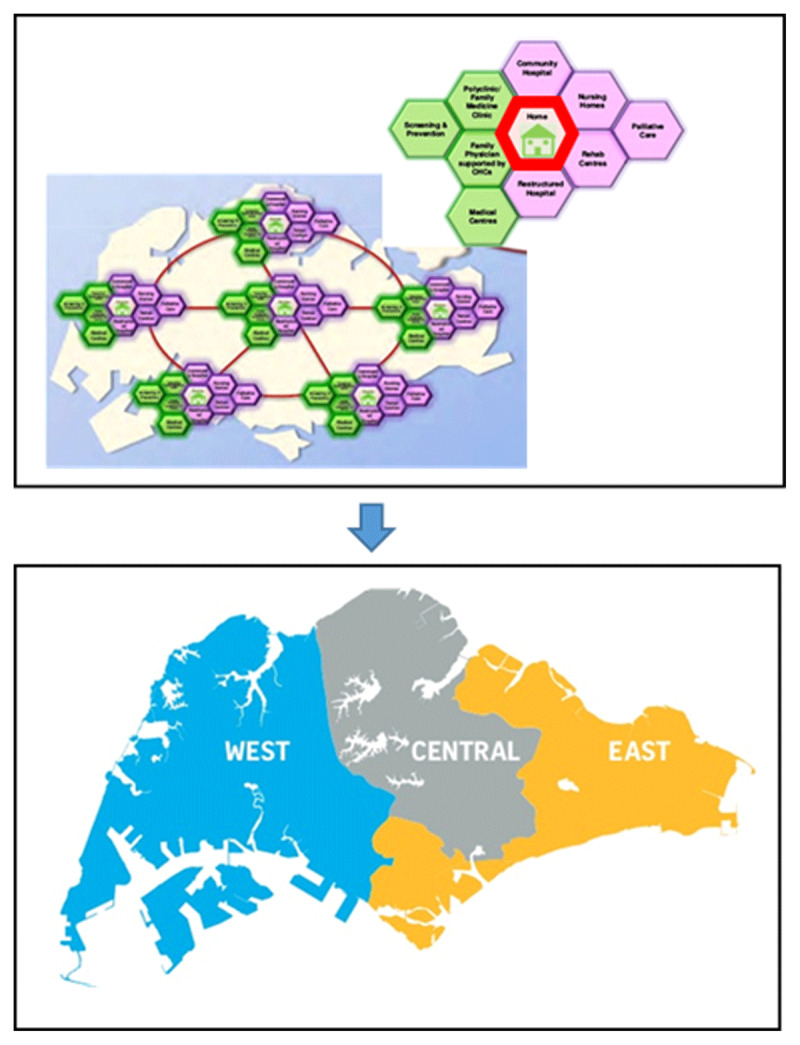
Re-clustering of six integrated regional health systems (RHSs) to three larger integrated RHSs in 2017 [8,9].

Health systems are facing challenges as the ageing population increases. From 2014 to 2017, the proportion of the Singaporean population aged 65 and above rose from 11.2% to 13.0% [1,2]. It has been projected that about 18.7% of its population would be aged 65 and above in 2030 [3]. The elderly were found to have more chronic conditions, with 80.6% of the age-group more than 65 years reporting one or more chronic diseases compared to 54.8% for that between 45 and 64 years [4]. With the increased health and social care needs of the elderly in Singapore, the healthcare system and policies have been evolving to embrace the principles of integrated care [5]. Singapore’s reforms started in 2000 when the government reorganised all polyclinics and restructured hospitals under two healthcare clusters–the National Healthcare Group (NHG) and Singapore Health Services (SingHealth)–to provide integrated care for patients [6]. From 2008 to 2009, six integrated regional health systems (RHSs) have been formed (***Figure 1***). Each RHS aimed to promote the integration of care services received by patients in public hospitals, primary care providers or intermediate and long-term care providers within a designated geographical area [6,7]. Further restructuring of the RHSs occurred in 2017, where three large RHSs were formed (***Figure 1***). While such re-clustering efforts aimed to promote greater integration of care, evidence on its benefits is still lacking [5].

**Figure 2:**
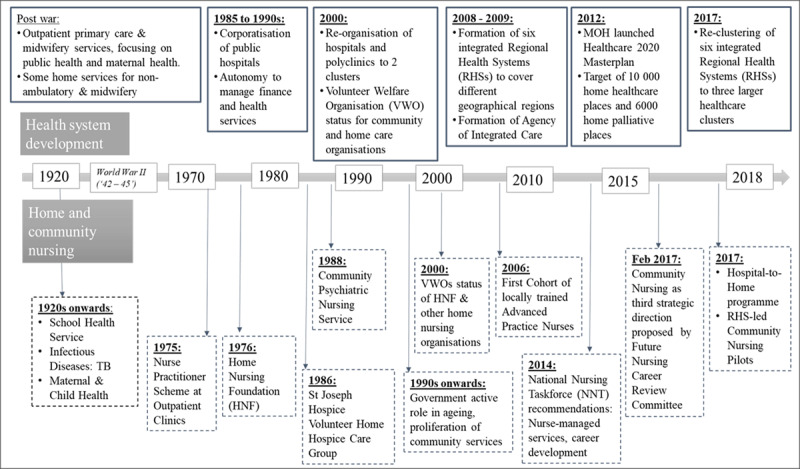
Development of Singapore’s healthcare system and home and community nursing [15].

With the need to provide holistic and coordinated care, nursing teams have evolved from a traditional flattened structure of nurses to multi-skilled and multi-professional teams [10]. The elderly often require coordination of care among multiple healthcare and social-care providers [11] and transitional care from the hospital to home [12]. This has also accentuated the role of nurses in care coordination and management [13,14]. Similar nursing developments have taken place in Singapore (***Figure 2***). The introduction of Hospital-to-Home (H2H) programme has driven the development of transitional care or RHS community nurses. These registered nurses (RN) were based in acute care hospitals and promote the safe and timely transfer of patients between care settings by taking on activities at hospital discharge and post-discharge care. This decreases preventable adverse events during care transition, such as medication errors and falls [12].

A literature review has been conducted to understand the changing roles of transitional care and community nurses in integrated healthcare systems. Common activities and practices performed by home and community nurses have been identified in previous studies [16,17,18]. They are direct patient care, patients’ monitoring, educational actions, psychosocial care and administrative work. There is also a shift away from the reactive nursing delivery system to models of care that focus on preventive home visits for older people, nurse-led post-discharge services and the specialisation of nursing work [19,20,21].

Transitional care programmes have been studied to identify the main interventions involved, duration of care and their effectiveness. In a recent scoping review, key professionals involved in transitional care teams were often nurses, with some of them receiving additional training in transitional care or speciality training [22]. Common interventions were discharge planning, medication management, structured needs assessment, patient education, chronic disease self-management, post-discharge guidance, caregiver support, care coordination and case management [12,23,24,25,26,27]. Delivery of transitional care varied in duration and frequencies of telephonic and/or home visitation mode [22,28].

Despite the programmes have demonstrated their usefulness and cost-effectiveness [23,29], the changing roles assumed by nurses were little explored. The programmes often focused on specific disease conditions or patient profiles [29,30] such that roles of the nurses could be seen as an extension of work settings from hospital to community. A recent study has demonstrated the interrelated influence of moderating factors on the implementation fidelity of the transitional care program [25]. Gaps in the literature could be seen in the overall change in the roles of nurses in the hospital-to-home interface and as an overall integrated system approach. Such understanding will be crucial in determining whether certain roles were assumed considering the changing contextual system factors and developments of other professional roles in this care system.

As integrated healthcare systems are increasingly formed, studies have shown their impact on the roles of nurses [31,32,33,34]. Coordination and partnership across settings and delegation and supervision of unlicensed personnel were reported [33]. There were also a high number of non-patient-related activities such as meetings, referrals and administrative tasks [31]. Although all of these studies have afforded some evidence on the influences of the integrated care system on the evolution of the nursing roles, the actual changes and development of these roles have not been well elucidated. A study to understand the changing roles of nurses is warranted. In particular, the drivers for the development of community and home nursing have remained less explored. This study aimed to examine the roles of transitional care nurses in an integrated RHS and the influence of the development of RHS on their evolving roles. The research questions were the following:

- What are the transitional care nursing roles in an integrated RHS?
- What are the various influencing factors in the integrated RHS on their roles?
- How does the development of the integrated RHS affect the change in the roles of transitional care nurses?

## Methods

### Study design

A qualitative case study research design was used to examine the influence of different contexts and social interactions on the nursing roles, offering insights into the complex interrelationships between the components [35,36].

### Theoretical framework

**Figure 3:**
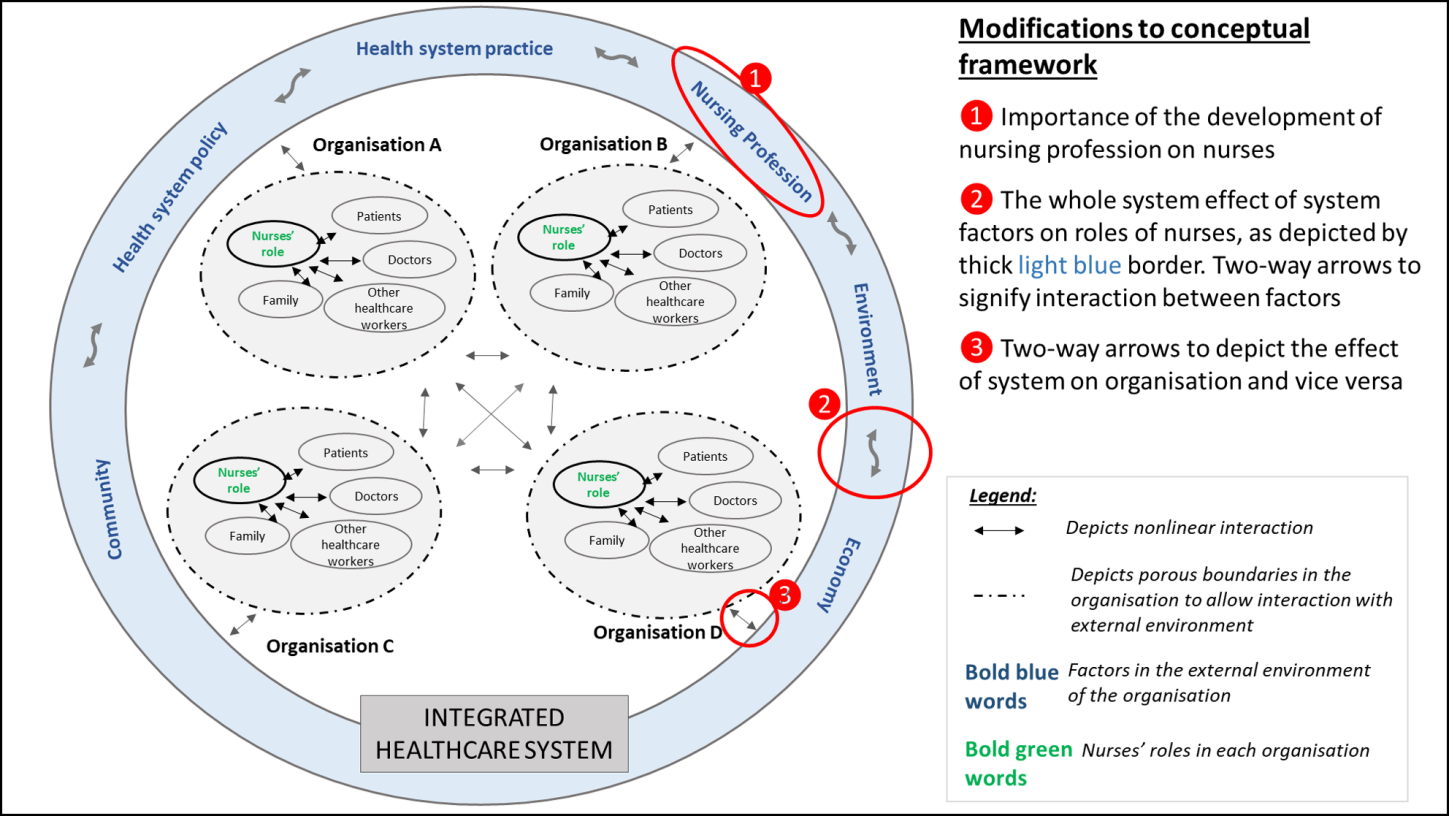
A refined conceptual framework that recognises the importance of relationships and interactions between different staff within each organisation and between different organisations [42,43].

The role theory and complex adaptive system (CAS) theory were used to guide the study [37,38]. A role can be defined as ‘the set of prescriptions defining what the behaviour of a position member should be’ [39] (p29). Three aspects of role can be defined, with reference to the person segment (role of a nurse), behaviour segment (nursing roles) and person–behaviour segment (patterns of behaviour characteristics of transitional care nurses). This role theory emphasised the importance of observation as the optimal method to gather the behaviours of the transitional care nurses. The construct of the observation charts and interview schedules have aimed to uncover the various roles taken on by these nurses. Previous researchers have also emphasised the dynamic properties of roles and various influences on the roles of nurses within a social system [37,40,41]. Drawing reference from two existing CAS conceptual frameworks in healthcare [42,43] and findings from a separate study in the first author’s thesis [15], the conceptual framework for this study is proposed. This framework (***Figure 3***) was based on the understanding that although nursing services are governed by systems of working within individual organisations, relationships were maintained between the organisations in the overarching integrated healthcare system. Besides emphasising the use of a qualitative case study research using multiple cases, this CAS framework guided the data analyses such that the within-case analysis preceded cross-case comparisons. The findings of the other three cases and the final analysis were reported separately in the first author’s thesis [15].

### Setting and sample

There were six integrated RHSs in Singapore before the mergers into three larger RHSs in January 2017. Despite the mergers, the operations in the six sub-systems have not changed drastically compared with their pre-merger times during data collection from April to September 2017. The sub-system of one of the three large RHSs in central Singapore was selected as the main research setting, hereafter referred to as ‘central RHS’. An instrumental case study was used to achieve the research objectives [35]. The transitional care team from the central RHS was the case of interest, named herein as ‘Case D’. This team formed a diverse case, offering a different insight to the roles of nurses providing care in a home environment [44]. Nurses in specially developed programmes to serve certain unique patient profiles, such as mental health and home ventilation, were excluded in this case.

Case D was managed under the main 1,500-bed tertiary hospital that anchored the central RHS. The hospital’s home-based care services had started with three silo programmes to serve post-discharge patients of different profiles: Aged Care Transition (ACTION), Virtual Hospital and Post-Acute Care at Home. The first programme aimed to provide case management service for patients with health and social care needs; the second programme targeted patients who were frequently admitted for chronic health conditions; and the third served to provide intensive medical and nursing care for clients with complex health care needs. The data collection was conducted after the merger of these three programmes into one transitional care team, allowing rich information to be collected. This team was led by nurses and supported by a multi-disciplinary team of doctors, pharmacists and allied health therapists in service provision. This service has also been financed by the H2H funding under the Ministry of Health since 2017. These RNs received in-house orientation and induction programme before they were merged into one department. They reviewed patients at their homes independently and work within escalation protocols for medical support through joint home visits and case discussions.

Purposive sampling was used to select participants. The inclusion criteria for the nurses were the following: (1) age of 21 years and above, (2) experience working as a nurse in transitional care services for more than 2 years and (3) provision of patient care for at least 24 hours a week to patients within the central RHS. The nurses were recruited for observations and follow-up interviews. Managers and other healthcare professionals (e.g. doctors, therapists and/or ancillary staff) were invited for interviews to elicit their perspectives on nurses’ work. Managers were included if they held key administrative positions in overseeing the department that governs nurses. The inclusion criteria for other healthcare professionals were the following: (1) age of 21 years and above, (2) working with a nurse participant and (3) provision of professional services under the organisation. Relevant documents, such as policies and forms that described the roles and practices of nurses in the last five years, were also examined.

### Ethical consideration

Ethical approval for the study was obtained from the Institutional Review Board of the RHS (Ref. No.: 2016/01418). The potential participants had first been identified by the nursing manager based on the inclusion and exclusion criteria. The study was explained to the prospective participants, and written informed consent was obtained by the first author. Anonymity and confidentiality were assured through the use of codes to identify the participants. Verbal consent to observe the nurses’ work was obtained from patients or caregivers prior entry to their homes.

### Data collection

Three data collection methods were used: non-participant observations of nurses by the first author, a registered nurse working in a tertiary hospital and doing her PhD project; individual interviews of nurses, managers and healthcare professionals; and a documentary analysis. Data collection continued to the point of data saturation, which was when the data set was completed and the research questions were answered [45]. The participants’ demographics were collected using self-completed demographic sheets.

Non-participant observations of the nurses during their regular working hours were undertaken for at least 24 hours a week. The first author asked them to provide care to patients as usual so that the data collected could genuinely reflect their practices. The first author followed each participant and observed their practices consecutively in a week. Two observation charts to document nurse-patient encounters during home visits and the nurses’ daily schedules were used to elicit the direct or non-direct care interventions and nursing work. Following the observations, the same nurses were interviewed individually based on a semi-structured interview guide to understand the ‘how’ and ‘why’ of the roles performed. Separate semi-structured interview guides were used for managers and healthcare professionals. All interviews were audio-recorded. Each interview lasted approximately 30–120 min. The interview guides were developed from the research questions and had been pilot-tested on a separate home hospice team, of which the findings were not used in this study. Lastly, documentary data were transcribed onto the data extraction form, which was constructed to capture relevant information on the work and roles of nurses [46].

### Data analysis

A simple descriptive analysis using Microsoft Excel 2013 was conducted on the quantitative data from the participants’ demographic sheets and daily observational information. Audio recordings of the interviews were transcribed verbatim, and handwritten observation notes were typed into electronic text. A thematic analysis was then undertaken for the qualitative data using the six-phase data analytical method [47], incorporated with techniques of data condensation, data display and conclusion drawing [48]. QSR Nvivo 10 was used to organise the data.

First, data were collected by the first author to allow familiarity of the data [47]. Next, the first and third authors independently generated the initial codes before checking for agreement [48]. During the third step in searching for themes, different codes were sorted into potential units of analysis to discern pattern codes [47,48]. In the fourth step, three authors refined the themes by examining the levels of the coded data extracts to ensure coherence in its patterns and consider individual themes in relation to the data set as a whole [47]. The themes were defined in step five before concluding with a written report in step six. Pattern matching and explanation building were employed in the within-case analysis [49].

### Methodological rigour

The four criteria of credibility, transferability, dependability and conformability were used to ensure the trustworthiness of the study [50]. Triangulation of the data collection methods and data sources was undertaken to enhance the credibility of the study [51,52]. Prolonged engagement was crucial to reduce the observer effect on the participants’ behaviours [53]. The authors undertook independent data analyses before coming together to ensure consistency in the coding and identification of themes [51]. The first author kept a research diary to enable the comprehension of the thoughts that led to the findings [51]. The study research design and findings are described in detail in the first author’s thesis [15] to allow for the transferability of the findings [54].

## Results

### Demographic data of the participants and observation information

A total of four nurses were selected based on the inclusion and exclusion criteria, and they covered different geographical regions served by the central RHS. Two managers and three healthcare professionals who worked closely with these four nurses were interviewed. ***Table 1*** presents the demographics of the participants. The nurse participants were observed over a total of 147 *h*ours over 17 days. Eleven home visits were made, with a mean duration of 54.55 *min*utes per home visit. A total of 11 relevant internal documents were reviewed here. These documents were mainly workflows, service manuals and induction materials.

**Table 1:** Demographics of the participants.


DEMOGRAPHICS	NURSES (N = 4)	HEALTHCARE PROFESSIONALS (N = 3)	MANAGERS (N = 2)

**Age (years)** (mean, range)	33.75 (29–37)	38.00 (^‡^)	42.5 (^‡^)

**Sex**			

Male	2	2	0

Female	2	1	2

**Race**			

Chinese	4	2	1

Malay	0	0	0

Indian	0	1	1

**Current job designation**	0 Staff nurse	Physiotherapist	Nurse clinician

	3 Senior staff nurse	Doctor	Nurse clinician

	1 Nursing officer	Assistant community care coordinator	

**Years of working in the current organisation** (mean, range)	9.50 (6–15)	4.16 (^‡^)	*


*Note*: ^‡^, range is not reported as the small numbers may reveal the identity of the participants; *, data not presented to ensure anonymity of the small number of participants.

### Themes and sub-themes of Case D: RHS hospital transitional care team

The themes and sub-themes are presented in ***Table 2***. The nurses’ roles arose because of the need to come together in the integrated healthcare system ‘to meet the needs of all’. Case D was at the centre of all change forces, and the nurses’ roles were developing to be ‘strong amidst the stormy waves’. The nurses’ roles were still evolving and ‘searching for the right formula to handle the complexity’ in the integrated healthcare system.

**Table 2:** Themes and sub-themes of Case D.


THEMES	SUB-THEMES

**Theme 1**: Coming together to meet the needs of all	• Rising out of the norm • Closing the gaps

**Theme 2**: Standing strong amidst the stormy waves	• Moving along with the storm of change • Shaking a solid foundation • Maintaining a distant relationship • Sustaining a lifeline in the storm

**Theme 3**: Searching for the right formula in handling complexity	• Facing complexity at its prime • Teaming with teams • Emerging differently


#### Theme 1: coming together to meet the needs of all

This theme describes the atypical roles undertaken by nurses in Case D (Sub-theme 1) and their attempts to close gaps and meet patients’ needs (Sub-theme 2).

##### Sub-theme 1: rising out of the norm

The observation of Case D nurses has demonstrated that the care delivered was beyond the traditional home visits, displaying the sub-theme of ‘out of the norm’. The nurses typically made only one visit for each patient as the first visit was considered free of charge for them. Therefore, during one single home visit, the nurses were observed to make a comprehensive health assessment, which included physical aspects, cognition, environmental safety, emotional well-being and social support system. As part of their role in care management, the nurses would promote adherence to chronic disease care plans by educating the patients and their caregivers. Ensuring medication adherence and reminding them of their appointments were significant components of their work. Coordination of care then followed to ensure that various health or social services were in place. Patients’ care management was executed mainly through telephone consultations.

Delivery of nursing care was not limited to direct contact with patients or caregivers at home visits or over the phone. The nurses were instrumental in discussing the patients with the multi-disciplinary team. A formal daily case discussion and weekly multi-disciplinary rounds (MDR) were held for each of the four sub-teams. Observations of the nurses also revealed that informal discussions with their multi-disciplinary team were common in the office.

*‘The nurse went to speak to the occupational therapist in the office regarding a home visit for the patient. The nurse arranged the timing for the visit and updated her about the case. The nurse also updated her regarding another case.’ [Observation–Nurse03]*

Proactive preventive care was observed as one of the nursing roles undertaken. The nurses received a national risk stratification list of hospitalised patients who might need services post-discharge. Each sub-team had a lead nurse to screen through the list and enrol patients with complex medical and social needs. As the service was developing, the nurses also participated in strategic development projects within the department as well as with other community providers.

##### Sub-theme 2: closing the gaps

There were unique features of the roles of the nurses, of which ‘closing the gaps’ was the most prominent observation. They made sure that the patients transited smoothly through various settings. Forming a safety net included addressing any new health concerns, providing health information and caregiver teaching and escalating rapidly to the medical team when these patients turned unstable. Although closing the gaps was the primary reason for the development of Case D, the nurses’ roles also addressed the current fragmentation of the integrated RHS.

*‘Because if we cannot cross that bridge right, then we try other bridge that can support. If there aren’t any bridge that can support. Then it will be due to a limitation in the service, which is something geographically or politically or service limitation wise, we cannot do anything about it. We should just try our best to help the patients.’ [Interview–Manager02]*

Closing the gaps has also meant that the nurses have moved away from the traditional manner of care delivery. Instead of solely functioning within a certain care setting, these nurses have worked in different settings such as inpatient wards, community settings and homes or via teleconsultation. The care network was expanded by their attendance at regular networking sessions and case discussions with the community providers and polyclinics (primary care). Although it was observed that the nurses spent much time on the telephone, these increased contacts have attempted to plug the gaps in the integrated RHS in which some patients had fallen through.

#### Theme 2: standing strong amidst the stormy waves

This theme has described how the nurses established their new roles in the face of various challenges, including the intense push for changes at the system and policy levels (Sub-theme 1), shaken foundation of the organisation (Sub-theme 2), remote influences of families (Sub-theme 3) and need to provide care to the most vulnerable patients (Sub-theme 4).

##### Sub-theme 1: moving along with the storm of change

The merger from the three programmes into one service was the most significant turning point for the nurses. This change was fuelled by the changing healthcare needs of the population. The participants echoed this, who explained that the patients were older and sicker and thus needed more care after hospitalisation. National programmes were introduced to expand the existing community healthcare services. Although the shift was welcomed, the nurses verbalised that the change in the funding structure has changed the care delivery. Programmes were nurse-led as the doctors’ visits were expensive. The free-of-charge nurse’s first visit was very intense to identify and address the patients’ biopsychosocial needs. One nurse also discussed her discomfort that her role was much determined by the cost of visits.

The governmental policies and funding on other primary and community services have exerted strong impacts on the nurses’ role. As the community services were run by non-for-profit organisations, different organisational sizes also meant different standards and capacities of the services. The disparate service capacities have resulted in some nurses holding on longer to their patients before handing them over to a suitable provider. Understanding the strengths and weaknesses of social services and working with them to improve their service scope has become part of their roles.

*‘The networking is with the community partners most of the time… … that helped us to come to a consensus that this is the part that you will do and this is the part that I will do. We can come together and synergise, and benefit the patients.’ [Interview–Manager01]*

##### Sub-theme 2: shaking a solid foundation

The expansion of the hospital outside of an institution setting has shaken its fundamental operating philosophy. The hospital was constantly setting new workflows, and regular briefing meetings were held. At times, there were uncertainties and confusion over the new and changing workflows by the team. Although the organisation has involved the nurses in the development of new workflows and processes, this also translated into heavier involvement in projects and meetings. The fast-paced changes have left nurses feeling overwhelmed.

*‘I think it is not from them [managers], it is from the top management that will sometimes shake the team a little bit. How come it is last minute? How come we are the last team to know? That kind of feel. It will be good that the management can prepare us in advance on what is going to happen.’ [Interview–Manager02]*

There was a shift in the model of care towards the nurse-led and team-based approach. One therapist shared that the doctors and therapists became consultative figures rather than directive ones. The shift in care delivery towards a team-based approach was evident. The managers, also known as the team leaders, have stood firm and resolute. They provided stability within the team by being the clinical support and by looking into nursing development to keep pace with rapid and constant changes. Standing firm amidst all of the changes and supporting the nurses in their roles were significant to maintain the cohesion of the team. They guided the nurses despite the lack of clarity.

*‘I just have to make the boat works. If not, the whole boat will collapse. I cannot demonstrate that I am shaking. I just have to keep it going.’ [Interview–Manager02]*

##### Sub-theme 3: maintaining a distant relationship

The relationship between the patients’ caregivers or families has exerted a lesser impact than other influences. Their main determining factor in establishing this relationship was often driven by the costs of the service. Even the enrolment and follow-up home visits were much decided by the family or caregivers. Regular updates and advice to family members and caregivers through telephone consults were commonly observed. The participants shared that these family members or caregivers have access to more health information via the internet. Communication via WhatsApp, text messages and emails were common. Because of the distant relationship, the nurses sometimes had little control of how the family members or caregivers managed the patients’ care.

*‘The nurse made a phone call to arrange a home visit as the son reported that the patient passed less urine. The nurse was concerned of urinary retention and was planning to do a bladder scan. However the son refused the home visit.’ [Observation–Nurse04]*

##### Sub-theme 4: sustaining a lifeline in the storm

The sub-theme ‘a lifeline in the storm’ describes the complex and challenging patients who depended on the nurses to prevent them from falling through gaps and weaknesses when the system has not integrated sufficiently to provide the care required by these patients. This particular group of patients often had extreme social circumstances or demonstrated non-adherence to lifestyle modifications or medications, leading to frequent disease exacerbations and hospital admissions. The patients’ multiple medical conditions sometimes limited the use of standard care plans. The patients had complex biopsychosocial needs such that several community services had to be in place. The nurses undertook the role as the single point of contact.

*‘One point of contact. By doing so, patients… by being one point of contact…… they don’t need to remember so many nurses’ names. And what we are dealing with are elderly, who tends to be more forgetful.’ [Interview–Nurse02]*

To meet the needs of these complex patients, the nurses first built a rapport with the patients. The nurses made contact with these patients in the wards before they were discharged and addressed their concerns when they encountered problems at home. One nurse described herself as ‘being a phone call away’. Thereafter, they would empower the patients in managing their own health. In a short transitional care period, they have ensured that patients were stable before handing over to a long-term care provider.

#### Theme 3: searching for the right formula in handling complexity

Case D has faced a number of complex changes as the RHS developed (Sub-theme 1) and working within a team and with several teams (Sub-theme 2). Their emerging roles in providing care differed from the norm (Sub-theme 3). Their evolving roles were still fraught with uncertainty, thus giving rise to the overarching theme of ‘searching for the right formula in handling complexity’.

##### Sub-theme 1: facing complexity at its prime

‘Complexity at its prime’ describes the pubescent stage of the changes in healthcare after the re-clustering in January 2017. The lack of awareness of other community services and their service capabilities was common. This was further hampered by the lack of system links between them, such that the nurses sometimes did not know the services received by the patients. The medical information documented by the community providers was not available on the National Electronic Health Records. Thus, the onus fell back on the nurses to communicate with the community providers to gather information. However, the constant and dynamic change was only the beginning as more upcoming changes were announced during the data collection period.

It is evident that the boundaries of work between different organisations and the rules in each organisation within the RHS have begun to change, leading to further complexity. It was observed that it was unclear whether certain nursing services were still within the scope. Protocols and workflows were continuously developed or modified. As the boundaries between the different organisations became blurred within the integrated system, there were duplications and gaps of services at times. The adaptability of the nurses came in useful when rules were unclear. In addition, the nurses were observed to display self-organising abilities and interacting within their own sub-teams and with other community providers. However, during such self-organisation and adaptation, one significant observation was that nurses unable to adapt effectively also departed from the system.

*‘Those [Nurses] who have eventually left. I would not say that because they cannot make it. It is because I think that there is something that they think it is not something for them.’ [Interview Nurse02]*

###### Sub-theme 2: teaming with teams

The nurses have formed a team that worked with several teams, thus giving rise to the sub-theme ‘team of teams’. Besides seeing the patients at home, they would assess the patients in the wards or clinics and hold case discussions with hospital teams or primary care teams. The nurses were recognised as the single source of contact and were supported by other healthcare professionals. Their role as a catalyst to other teams was also observed. In the community, they frequently collaborated with other homecare nurses and primary care and community providers. For ways to connect with them, other than the face-to-face physical presence in the wards or clinics, formal methods included holding joint MDR or teleconsultations. For more complex patients, joint home visits were made. The nurses usually followed up by speaking to the staff to discuss the patients after these formal communications. The various ways of communicating with several teams emphasised the role of nurses as good communicators.

*‘My efforts might be limited by one self. Let’s say I gather a team of community partners. Together with community partners, working with them, to let them know that there is a shared common patient goal, then they help with whichever means of expertise. [Interview–Nurse03]*

###### Sub-theme 3: emerging differently

As the hospital developed its community services in tandem with the integrated healthcare system, new roles were emerging. Because of the focus on integration and collaboration, the ways through which the nurses conducted their usual assessment have changed. Possible enrolment into the service was no longer only referred but was identified by a national risk stratification tool. These patients had to be assessed proactively to look for any unidentified needs and refer appropriately to the various community services.

*‘As the patients’ conditions get more complex, the complex care does not allow the nurses to be so hospital-based, clinic-based. Rather be more proactive. Proactive to go in and be more engaging with the patients……. To elicit the behavioural changes in getting well.’ [Interview–Nurse03]*

The drive towards integrated care has meant that the nurses have to coordinate with both the specialists and community partners. Working in such a grey zone has meant that their caseloads were always shared. In this multi-prolonged integrative work, the physical presence and contacts of the nurses could be observed at different parts of the integrated RHS. The observations had shown that their roles were required to cover the current gaps in the integrated RHS when other providers were unable to provide the services on time.

## Discussion

The research aimed to understand the changing roles of transitional care nurses in the integrated healthcare system. Key insights were gained on their roles, the systems in which they worked and the evolution of their roles as the integrated RHS developed.

Diverse nursing roles have been reported in the literature as new community programmes and initiatives were implemented to facilitate the transition of care between settings [12,19,20,21,23,24,25,26,27,33]. The health assessment, telephonic support, coordination and chronic disease management roles were similarly performed by nurses in Case D. Proactive recruitment was also in place to identify at-risk patients using predictive tools and early institution of preventive measures. The literature has also witnessed this increasing shift of roles from passive and reactive to proactive care [20,21,33]. Emphasis was also placed on discharge planning and coordinating with other professionals to prepare patients and caregivers for their post-discharge care [12]. More studies should be conducted on the importance of this anticipatory and integrative role by transitional care nurses to ensure other care partners to take over patients’ care when they bridge from hospital to community.

The findings have shown that the nurses in Case D have extended from clinically-focused roles and holistic direct patient care activities to other new roles in networking, project planning and representation in workgroup and committees. A ‘rising out of the norm’ may be the new working philosophy to place nurses as key players in developing integrated care. The nurses’ roles in Case D were unique when they were ‘closing the gaps’ through their provision of interim nursing interventions, using different forms of care delivery and working in different care settings. Nurses often bridged the gaps and worked between boundaries of health and social care [55]. In addition, Case D nurses have taken on the system roles in working with care partners to close the gaps through the development of collaborative partnerships and were beyond the usually described direct patient-nurse care in literature [12,23,24,25,26,27].

The findings have highlighted the impact of the restructuring of the healthcare system towards community care on the roles of nurses. Studies have similarly outlined the possible socio-political influences on nurses’ roles [56,57]. Findings have shown that nurses played a role not only in their organisations but also in the integrated RHS by partaking in collaborative meetings and developing services and workflows with other community partners. It was evident that these new and expanded roles of the transitional care nurses have been introduced as a result of the change in the funding system for various programmes. Although such increased funding has been welcomed to drive the rapid shift to community care, Schofield et al. (2011) have cautioned that community programmes were often suspended when competing demands for funds emerged [58]. Confusion created by the lack of well-developed program direction and protocols was similarly observed in other national transitional care programme [25]. Such policy and system changes have to be managed carefully so as not to place nurses in an uncertain state of change.

The presence of nursing managers and leaders was significant in fostering the growth and development of the roles of nurses in the integrated care system. A qualitative study has shown that leadership in community nursing was crucial in how policies are delivered and how leaders have translated the policies into action plans for frontline community nurses to deliver care [59]. In this study, the team leaders concurrently managed the senior management upstream and nurses in their teams downstream. The effect of the patterns of the patients’ family and caregivers on the roles of nurses is noteworthy. Similar to other Asian studies, family members were heavily involved in medical decision-making [60], and the hired foreign domestic workers and themselves were the direct care providers in home settings [61]. Although cost was often stated not as a barrier for patients to receive care [25], negotiations with the family to provide services have fallen on the nurses who have to balance between professional obligations and the family’s financial concerns.

The development of the integrated RHS on nursing roles and possible mechanisms of change were examined through the lens of complexity science and the role theory. The changes in the roles were largely complex, even though some form of structure and order was present. CAS theorists have highlighted that the organisational structures and processes have facilitated informal exchanges and interactions [62,63,64]. This research has demonstrated that the nurses have expanded linkages throughout the integrated RHS with a high amount and level of interactions undertaken. Although it may appear chaotic, there were actually ‘order within chaos’ as the nurses self-organised their work towards integrated care [65].

Adaptable and self-organising attributes of the nurses have been observed to ‘emerge differently’ in this new complex integrated care system. The roles of the nurses in various programmes in Case D were merged as one, and new roles were introduced. It was observed that establishing new nursing roles was also a dynamic process as the integrated healthcare system develops. Although it is tempting to reduce complexity and ensure certainty by managing persons and creating structures [66], the findings have suggested that broad frameworks and healthcare policies should be provided to allow local adaptability yet prevent wide variations in the roles of nurses [43,67]. In addition, the processes should only be formalised when necessary: this will avoid having several prohibitive workflows and formal guidelines that add to administrative work but serve little clinical purpose.

The strengths of this study were that this research took place when the RHS underwent organisational changes. It is also noteworthy that this study has afforded valuable insights into nurses who provided care at home in an Asian context. The findings have also informed the importance of transitional nursing interventions in integrated care systems, and these roles should be further emphasised in the national community nursing scope of practice and development. This study also revealed the significance of organisational structures and policies in influencing the roles of transitional care nurses. Adequate support in terms of educational preparation and continued expansion of their roles should be undertaken.

**Figure 4:**
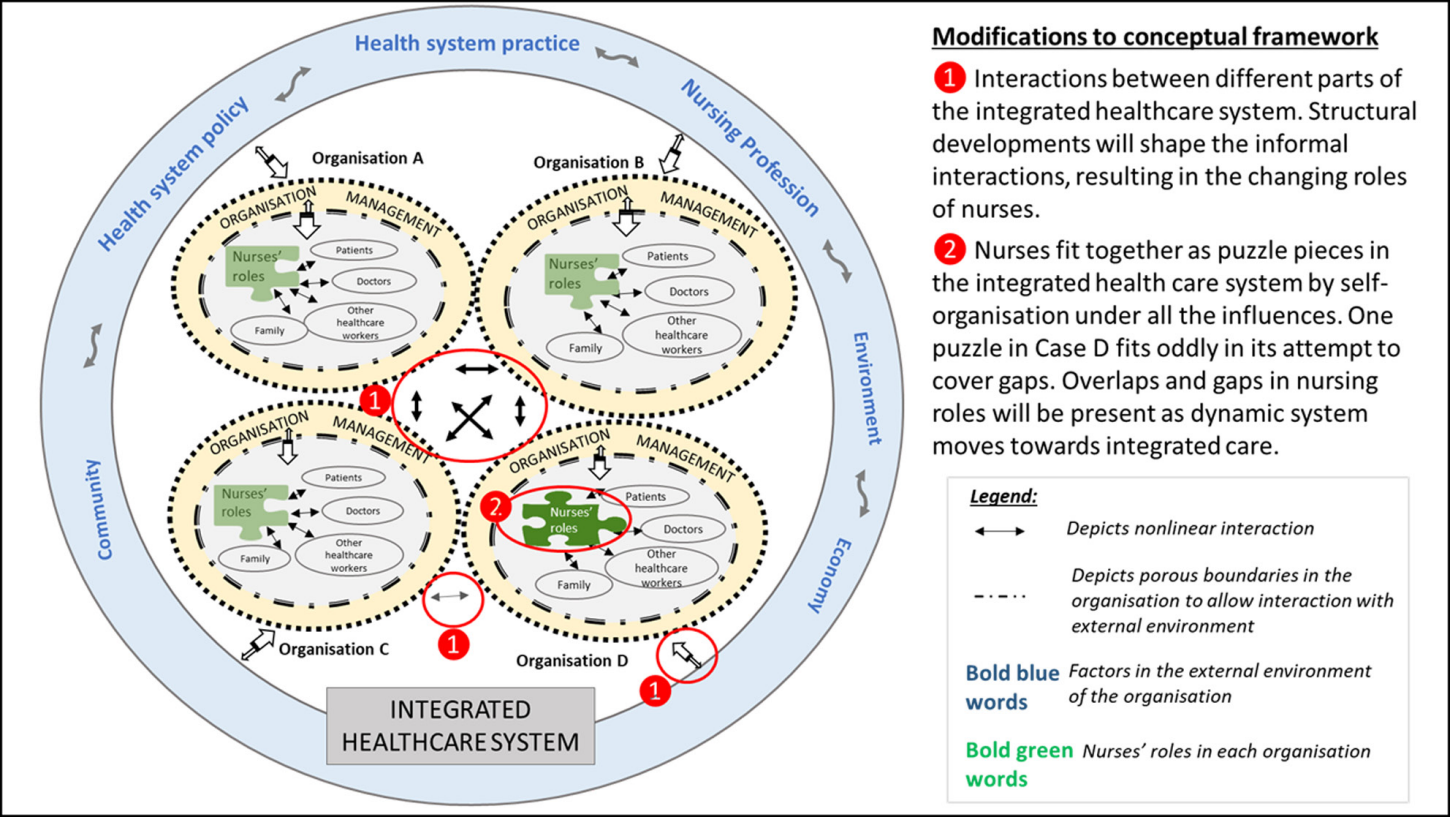
Proposed conceptual framework of the changing homecare nursing roles in an integrated regional health system (RHS).

Although the limitation of generalisation due to the small number of nurses is present, a longer observation period of over a week per nurse has been undertaken. Although it is recognised that the findings reported here might be limited to a single case study, the first author has conducted three similar case studies to provide the cross-case comparison in her final thesis and further modified the CAS conceptual framework (***Figure 4***). More studies are recommended using the framework, given the complexity of healthcare services and systems. Lastly, it is recommended that future studies on nursing roles should include observation as a data collection method instead of solely depending on interview methods as this method allows accurate capturing of multiple roles.

## Conclusion

The study has demonstrated the shift in the roles of transitional care nurses as the healthcare system grows increasingly complex and the replacement of linear thinking models with complexity science. The evolvement of the roles of nurses will be continuous and dynamic as different influencing factors come together and interact at varying strengths. The findings have contributed to developing the conceptual framework, which will enhance understanding the shift of nurses roles as the integrated care system develops. Further studies on the evolvement of nursing roles as the integrated RHS develops are strongly recommended to provide insights to future policy designs and nursing profession advancement.
